# 

**Published:** 1903-06-13

**Authors:** 


### NOTICE TO CORRESPONDENTS.

All MSS., Letters, Books for Review, and other matters intended for the Editor should be addressed The Editor, "The Hospital," The Hospital Buildings, 28 & 29 Southampton Street, Strand, W.C.

The Editor cannot undertake to return rejected MS., even when accompanied by stamped directed envelope.

**Notice to Advertisers and Subscribers.**

All Advertisements, Orders for Copies of The Hospital, and Business Communications should be addressed to **THE MANAGER, (Not to the Editor)**, The Hospital, 28 & 29 Southampton Street, Strand, London, W.C.

The Cost of Subscription, post free, is as follows.—Per week, 3½d.; per quarter, 3s. 9d.; per half-year, 7s. 6d.; per annum, 15s. Foreign and Colonial:—Per annum, 19s. 6d., payable, in all cases, in advance.

**NOTICE.—Vol. XXXIII. of "The Hospital" (October 1902 to March 1903) in handsome cloth binding, will shortly be on sale at the office of this Journal, price 7s. 6d. Cases for binding the Numbers contained in this Volume 1s. 6d. Index to Vol. XXXIII., 2½d., post free.**

**The monthly part for June is now ready. Price 1s., by post 1s. 3d.**

### THE STUDENT OF MEDICINE.

### APPOINTMENTS.

**W. N. Barron**, M.R.C.S., L.R.C.P.Lond., appointed Medical Officer for the Winkfield District of the Easthampstead Union. **J. A. T. Cartwright**, M.R.C.S., L.S.A., L.M., reappointed Medical Officer of Health for Wigmore Rural District. **Frank Colman**, L.D.S., appointed Dental Surgeon to the East London Hospital for Children, Shadwell. **George Cuscaden**, L.R.C.P.&S.Edin., appointed Honorary Surgeon to Out-patients at the Women's Hospital, Melbourne. **G. W. Dick**, L.R.C.P.&S.Edin., appointed Certifying Factory Surgeon for the Shotts District, Lanarkshire. **A. A. Edward**, M.D.Edin., C.M., appointed Certifying Factory Surgeon for the Haslingden District, Lancaster. **Charles W. Forsyth**, M.R.C.S., L.R.C.P., appointed Assistant Medical Officer to the St. Mary's Islington Infirmary, Highgate Hill. **Janet Greig**, M.B., Ch.B.Melb., appointed Honorary Assistant Anæsthetist to the Melbourne Hospital. **Alfred Grimwade**, M.B., Ch.B.Melb., appointed Honorary Assistant Anæsthetist to the Melbourne Hospital. **Reginald Hawden**, M.B., Ch.B.Melb., appointed Resident Medical Officer of the Warrnambool Hospital, Victoria. **Charles Holmes Joy**, M.D., B.S.Durh., appointed Certifying Factory Surgeon for the Tamworth District of Staffs. **Charles Frederick Knox**, L.R.C.P.Edin., M.R.C.S.Eng., appointed District Medical Officer of Port of Spain, and Medical Inspector of Immigrants in Trinidad. **J. M. McGill**, M.B., C.M.Edin., appointed Certifying Factory Surgeon for the Annbank District, Ayrshire. **H. B. Morison**, M.B., appointed District Medical Officer of the Bromley Union. **Frank Pershouse**, M.R.C.S., L.R.C.P.Lond., appointed District Medical Officer of the Orsett Union. **A. Philip**, M.D., appointed Certifying Factory Surgeon for the Newport District, Fifeshire. **W. A. Reid**, L.R.C.P.&S.Edin., appointed Junior House-Surgeon at the Clayton Hospital and Wakefield General Dispensary. **Arthur Edmund Shepherd**, L.R.C.P., L.R.C.S., appointed Honorary Gynæcologist to the Adelaide Hospital. **Wm. Sinclair**, M.B., C.M.Aberd., appointed Medical Superintendent of the Aberdeen Royal Infirmary. **Arnold J. Thomson**, M.R.C.S., L.R.C.P.Lond., appointed Medical Officer to the Stourbridge Union Workhouse. **W. W. Wood**, M.B., C.M.Edin., appointed Certifying Factory Surgeon for the Grangemouth District, Stirlingshire.

ST. THOMAS'S HOSPITAL.—The following gentlemen have been selected as House Officers from Tuesday, June 2nd, 1903:—House Pysicians: **C. N. Sears**, L.R.C.P., M.R.C.S. (*Extension*); **A. E. Boycott**, M.A., M.B., B.Ch.Oxon, B.S.Oxon. (*Extension*); **O. Hildesheim**, B.A., M.B., B.Ch.Oxon; **H. W. Sexton**, L.R.C.P., M.R.C.S. Assistant House Physicians: **B. N. Panton**, B.A. Cantab., L.R.C.P., M.R.C.S. **J. N. Sergeant**, L.R.C.P., M.R C S. House Surgeons (*Extensions*): **J. W. Rob**, B.A., M.B., B.C.Cantab.; **T. B. Henderson**, M.A., M.B., B.Ch.Oxon.; **J. P. Hedley**, M.A., M.B., B.C.Cantab.; **A. B. Bradford**, M.B., B.S.Durh., L.R.C.P., M.R.C S. Assistant House Surgeons (*Extensions*): **J. E. Adams**, L.R.C.P., M.R.C.S.; **H. Upcott**, L.R C.P., M.R.C.S.; **C. Wheen**, B.A.Oxon., L.R.C.P., M.R.C.S.; **N. Carpmael**, L.R.C.P., M.R.C.S. Obstetric House Physicians: (*Senior*) **W. M. G. Glanville**, B.A., M.B., B.Ch.Oxon. (*Junior*) **H. Spurrier**, B.A.Cantab., L.R.C.P., M.R C.S. Clinical Assistants: (Throat) **R. E. H. Leach**, B.A.Oxon., L.R.C.P., M.R.C.S. (*Extension*); **J. Coates**, L.R.C.P., M.R.C.S (Skin) **T. Guthrie**, B.A.Cantab., L.R.C.P., M.R.C.S. (*Extension*); **W. Ibbotson**, L.R.C.P., M.R.C.S. (Ear) **A. Bevan**, M.B.Lond., L.R.C.P., M.R.C.S. (*Extension*); **E. A. Ross**, M.B., B.C.Cantab.

### "The Hospital" Institutional Library.

"Hospitals and Asylums of the World," 4 Vols., with a Portfolio of Plans, complete £12 12s.

"Burdett's Hospitals and Charities, 1902." 5s.

"Burdett's Official Nursing Directory, 1903." 3s. net.

"Cottage Hospitals, General, Fever, and Convalescent." 10s. 6d.

"Uniform System of Accounts for Hospitals and Public Institutions." New Edition. 4s. net.

"Hospital Expenditure: The Commissariat." 2s. 6d.

"A Legal Handbook for the Use of Hospital Authorities." 2s. 6d.

"The Cottage Hospital Case Book or Register of Patients." 10s. and 12s. 6d.

"The Furnishing and Appliances of a Cottage." 6d.

All these are published by the SCIENTIFIC PRESS, Ltd., and may be obtained through any bookseller, or direct from the publishers, 28 and 29 Southampton Street, Strand, London, W.C.

### The Probationer's Library.

### THE NURSING PROFESSION:

### HOW AND WHERE TO TRAIN.

**Edited by SIR HENRY BURDETT, K.C.B.**

An Indispensable Guide to the would-be Nurse. Price **2/-** net; **2/4** post free.

### Three Admirable Nursing Handbooks.

### A HANDBOOK FOR NURSES.

By Dr. J. K. Watson. Price **5/-** net; **5/4** post free.
Very full and up to date.

### NURSING: ITS THEORY AND PRACTICE.

By Dr. Percy G. Lewis. Price **3/6** post free.
A complete Guide for the Medical, Surgical and Monthly Nurse.

### NURSING: ITS PRINCIPLES AND PRACTICE.

By Isabel Hampton. Price **7/6** net; **7/10** post free.
A valuable Guide to Nursing by a well-known American Matron.

### ELEMENTARY ANATOMY AND SURGERY FOR NURSES.

By W. McAdam Eccles, F.R.C.S. Price **2/6** post free.
A clear and concise instructor.

### ELEMENTARY PHYSIOLOGY FOR NURSES.

By Dr. C. F. Marshall. Price **2/-** post free.
The subject is completely and clearly explained.

### THE NURSE'S DICTIONARY OF MEDICAL TERMS AND NURSING TREATMENT.

Compiled by Honnor Morten. Price **2/-** post free.
An invaluable pocket-book for Nurses.

*These books are obtainable of all Booksellers and are published by* THE SCIENTIFIC PRESS, LIMITED, 28 & 29 Southampton Street, Strand, London, *who will send their latest Catalogue of Nursing Text-books post free to any Nurse on application.*

